# CRISPR-Mediated Transcriptional Repression in Toxoplasma gondii

**DOI:** 10.1128/mSphere.00474-21

**Published:** 2021-10-13

**Authors:** Benedikt M. Markus, Elizabeth A. Boydston, Sebastian Lourido

**Affiliations:** a Whitehead Institute for Biomedical Research, Cambridge, Massachusetts, USA; b Faculty of Biology, University of Freiburg, Freiburg, Germany; c Department of Biology, Massachusetts Institute of Technology, Cambridge, Massachusetts, USA; University at Buffalo

**Keywords:** CRISPR interference, CRISPRi, dCas9, knockdown, genome engineering, parasite, Apicomplexa

## Abstract

Tools for tuning endogenous gene expression are key to determining the genetic basis of diverse cellular phenotypes. Although synthetic regulatable promoters are available in *Toxoplasma*, scalable methods for targeted and combinatorial downregulation of gene expression—like RNA interference—have yet to be developed. To investigate the feasibility of CRISPR-mediated transcriptional regulation, we examined the function of two catalytically inactive Cas9 (dCas9) orthologs, from Streptococcus pyogenes and Streptococcus thermophilus, in *Toxoplasma*. Following the addition of single-guide RNAs (sgRNAs) targeting the promoter and 5′ untranslated region (UTR) of the surface antigen gene *SAG1*, we profiled changes in protein abundance of targeted genes by flow cytometry for transcriptional reporters and immunoblotting. We found that the dCas9 orthologs generated a range of target gene expression levels, and the degree of repression was durable and stably inherited. Therefore, S. pyogenes and S. thermophilus dCas9 can effectively produce intermediate levels of gene expression in *Toxoplasma*. The distinct sgRNA scaffold requirements of the two dCas9s permit their orthogonal use for simultaneous examination of two distinct loci through transcriptional modulation, labeling for microscopy-based studies, or other dCas9-based approaches. Taking advantage of newly available genomic transcription start site data, these tools will aid in the development of new loss-of-function screening approaches in *Toxoplasma*.

**IMPORTANCE** Toxoplasma gondii is a ubiquitous intracellular parasite of humans and animals that causes life-threatening disease in immunocompromised patients, fetal abnormalities when contracted during gestation, and recurrent eye lesions in some patients. Despite its health implications, about half of the *Toxoplasma* genome still lacks functional annotation. A particularly powerful tool for the investigation of an organism’s cell biology is the modulation of gene expression, which can produce the subtle phenotypes often required for informing gene function. In *Toxoplasma*, such tools have limited throughput and versatility. Here, we detail the adaptation of a new set of tools based on CRISPR-Cas9, which allows the targeted downregulation of gene expression in *Toxoplasma*. With its scalability and adaptability to diverse genomic loci, this approach has the potential to greatly accelerate the functional characterization of the *Toxoplasma* genome.

## INTRODUCTION

The protozoan parasite Toxoplasma gondii is an obligate intracellular pathogen of the phylum Apicomplexa, which includes many other important pathogens of humans and livestock, including *Plasmodium* and *Cryptosporidium* spp. Despite their global health importance, few species have been studied in molecular detail, and fewer than half of their genes have been functionally annotated. Previous adaptations of the CRISPR-Cas9 system have greatly facilitated functional genomics in these organisms (1–3) (for a recent review, see reference 4). Recently, we used genome-wide CRISPR-mediated gene disruption screens to identify *Toxoplasma* genes required for parasite growth in human fibroblasts (5). Such gene knockout approaches can readily assess gene essentiality; however, they fail to capture intermediate phenotypes that can result from partial loss of gene function due to dosage sensitivity, which may be distinct from the complete loss of the gene in question. Generating such phenotypes can be achieved by tuning gene expression via knockdown approaches, enabling a finer dissection of gene function (6).

In the absence of an exploitable system for RNA interference in *Toxoplasma*, knockdown approaches have been limited to the use of synthetic tetracycline-inducible promoters (7), as well as systems that rely on the destabilization of the targeted mRNA (8) or protein (9, 10). However, these methods are laborious and difficult to scale and necessitate modification of the target locus. In other biological systems, adaptations of CRISPR-Cas9 have given rise to technologies that enable the transcriptional tuning of genes without requiring genome editing. These tools exploit the ability of dead Cas9 (dCas9), a mutated Cas9 protein lacking endonuclease activity, to act as an RNA-guided DNA-binding protein (11, 12). Targeted to the promoter of a gene of interest using a single-guide RNA (sgRNA), dCas9—most commonly derived from Streptococcus pyogenes (*Spy*.dCas9)—can prevent the assembly or progression of the transcriptional machinery resulting in gene knockdown. Such a CRISPR interference (CRISPRi) system has the advantage of being broadly applicable to both protein-coding and noncoding RNA genes in their native context, inducing specific and reversible knockdowns. CRISPRi has been shown to be effective in prokaryotes and eukaryotes (11), including *Plasmodium* spp. (13–16). The approach is easily scalable and amenable to pooled genetic screens. Indeed, CRISPRi has been used extensively in bacteria and mammalian cell lines to study mechanisms of drug resistance and map gene interaction networks (17, 18; reviewed in reference 19). Such targeted approaches for transcriptional repression at a multigene scale are powerful functional genomics tools yet are lacking in *Toxoplasma*.

Recent studies in Plasmodium falciparum and Plasmodium yoelii adapted CRISPRi for the study of essential genes and successfully generated nonlethal phenotypes that could be used to elucidate gene function. While CRISPRi in mammalian systems most commonly employs a *Spy*.dCas9 fusion with the KRAB repressor domain to further enhance repression, such transcriptionally repressive domains have not been adequately defined in apicomplexans. Using *Spy*.dCas9 alone achieved moderate repression levels of the examined protein-coding genes in P. yoelii (up to 3.1-fold) (13) and in P. falciparum (up to 1.7-fold) (14). Another study in P. falciparum demonstrated the simultaneous knockdown (up to 11-fold) of an entire noncoding RNA gene family by targeting a conserved region (16). The fusion of *Spy*.dCas9 to the *Pf*GCN5 histone acetyltransferase and the *Pf*Sir2a histone deacetylase has recently also enabled transcriptional regulation via epigenomic editing in P. falciparum, resulting in the down- or upregulation of gene expression of target genes, respectively (15). In addition to approaches for transcriptional and epigenetic manipulation, *Spy*.dCas9 has also been adapted for the targeted coimmunoprecipitation of specific genomic loci in P. falciparum—a technique that allows the identification of factors associated with a genomic region of interest (20). While the potential applications for dCas9 in the study of apicomplexan genomes are numerous, further developments are needed to achieve the same level of precision and scalability that is already available in mammalian systems.

Here, we adapted CRISPRi for transcriptional repression in *Toxoplasma*. To assess changes in protein expression associated with the binding of dCas9 to promoter regions, we built a fluorescent reporter strain in which expression of mNeonGreen was driven by a copy of the upstream region of the surface antigen 1 gene (*SAG1*). Expressing *Spy*.dCas9 combined with individual sgRNAs targeting the *SAG1* upstream region induced modest repression levels (median up to 5.6-fold) at the reporter locus. The different repression levels appeared to be largely sgRNA position dependent (but strand independent), highly reproducible, and stable over time. The native *SAG1* locus, however, appeared to be less responsive to repression by *Spy*.dCas9 binding. Seeking alternative systems with improved potency for CRISPRi, we found that a dCas9 ortholog from S. thermophilus (*Sth*.dCas9) may be more robust in repressing transcription at the native *SAG1* locus. We note that more comprehensive comparisons between the two dCas9 orthologs and systematic testing of larger sgRNA sets will be necessary to fully elucidate the rules and characteristics of dCas9 repression. Our work shows that two dCas9 orthologs can modestly reduce gene expression in *Toxoplasma* when paired with an appropriate sgRNA, even without the fusion of repressive domains. By demonstrating CRISPRi via two orthogonally compatible dCas9s, this study provides an expanded repertoire of tools for transcriptional manipulation and other dCas9-based approaches in *Toxoplasma*.

## RESULTS

### Generating a stable S. pyogenes dCas9-expressing parasite strain.

**FIG 1:**
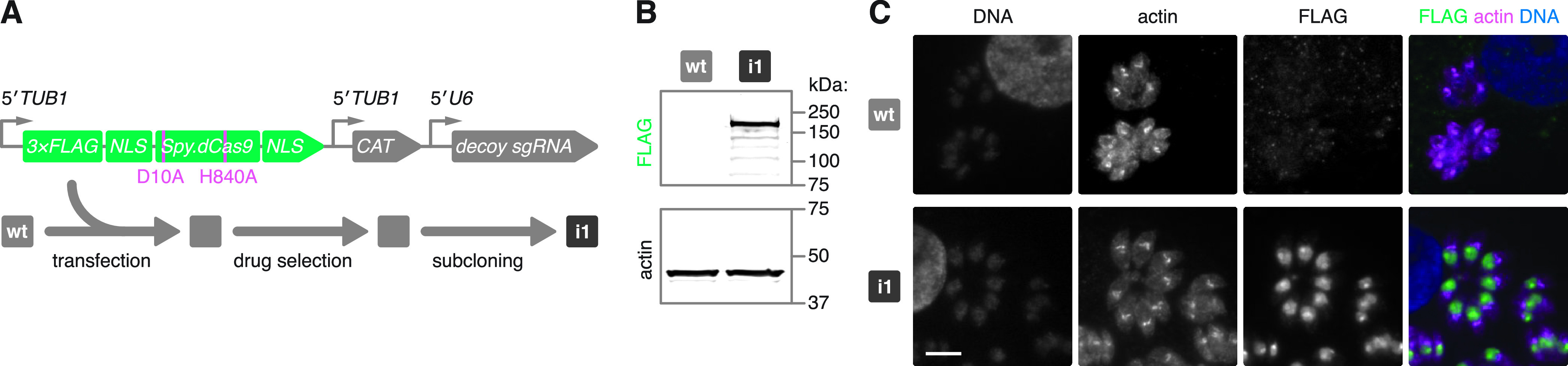
Generating a stable S. pyogenes dCas9-expressing parasite strain. (A) Expression construct and strategy for generating a clonal *Spy*.dCas9-expressing strain. *Spy*.dCas9 was expressed with an N-terminal FLAG tag. The coexpressed “decoy” sgRNA targets downstream of the endogenous *NHE1* ORF and was previously shown to mitigate toxicity caused by the heterologous expression of *Spy*.Cas9 (5, 21). Subsequent to the design of this construct, it was shown that the decoy sgRNA does not improve *Spy*.dCas9 expression (20). Following transfection and chloramphenicol selection of this construct in wt parasites, the resulting population was subcloned by limiting dilution to isolate CRISPRi strain 1 (i1). CAT, chloramphenicol acetyltransferase. (B) Immunoblots probing for FLAG and parasite actin. The expected molecular weight of FLAG-tagged *Spy*.dCas9 is 165 kDa. Actin served as a loading control. (C) Representative immunofluorescence images of formaldehyde-fixed intracellular parasites. Cells were stained for FLAG. Parasite actin provided a cytosolic parasite stain, and Hoechst was used to stain host and parasite DNA. Bar, 5 μm.

We sought to use CRISPRi with *Spy*.dCas9 alone to sterically impede the association of RNA polymerase II or other transcription factors at a gene’s promoter region. Previous work showed that the generation of *Toxoplasma* cell lines stably expressing *Spy*.Cas9 was facilitated by coexpression of a “decoy” sgRNA (5). Based on these findings, we devised a construct carrying expression cassettes for both a specific sgRNA and *Spy*.dCas9. It was only subsequent to the design of this construct that we found no improvement in *Spy*.dCas9 expression by the coexpressed sgRNA (21). Cas9 nuclease activity was inactivated by point mutations (D10A and H840A) within its HNH and RuvC nuclease domains (11, 22) (Fig. 1A). We transfected this construct into the canonical type I RH strain of *Toxoplasma*, which is commonly used in cell culture and which we refer to here as wild type (wt). Upon drug selection for stable integration of the expression construct into the parasite genome, we isolated a clonal population, which we refer to as CRISPRi strain 1 (i1). With this strain, we observed expression of full-length FLAG-tagged *Spy*.dCas9 by immunoblotting (Fig. 1B) and nuclear localization of the transgene by immunofluorescence microscopy (Fig. 1C). Lower FLAG-positive protein bands were also present in the blot, which may correspond to degradation products of *Spy*.dCas9.

### S. pyogenes dCas9 generates intermediate repression levels with high reproducibility.

**FIG 2:**
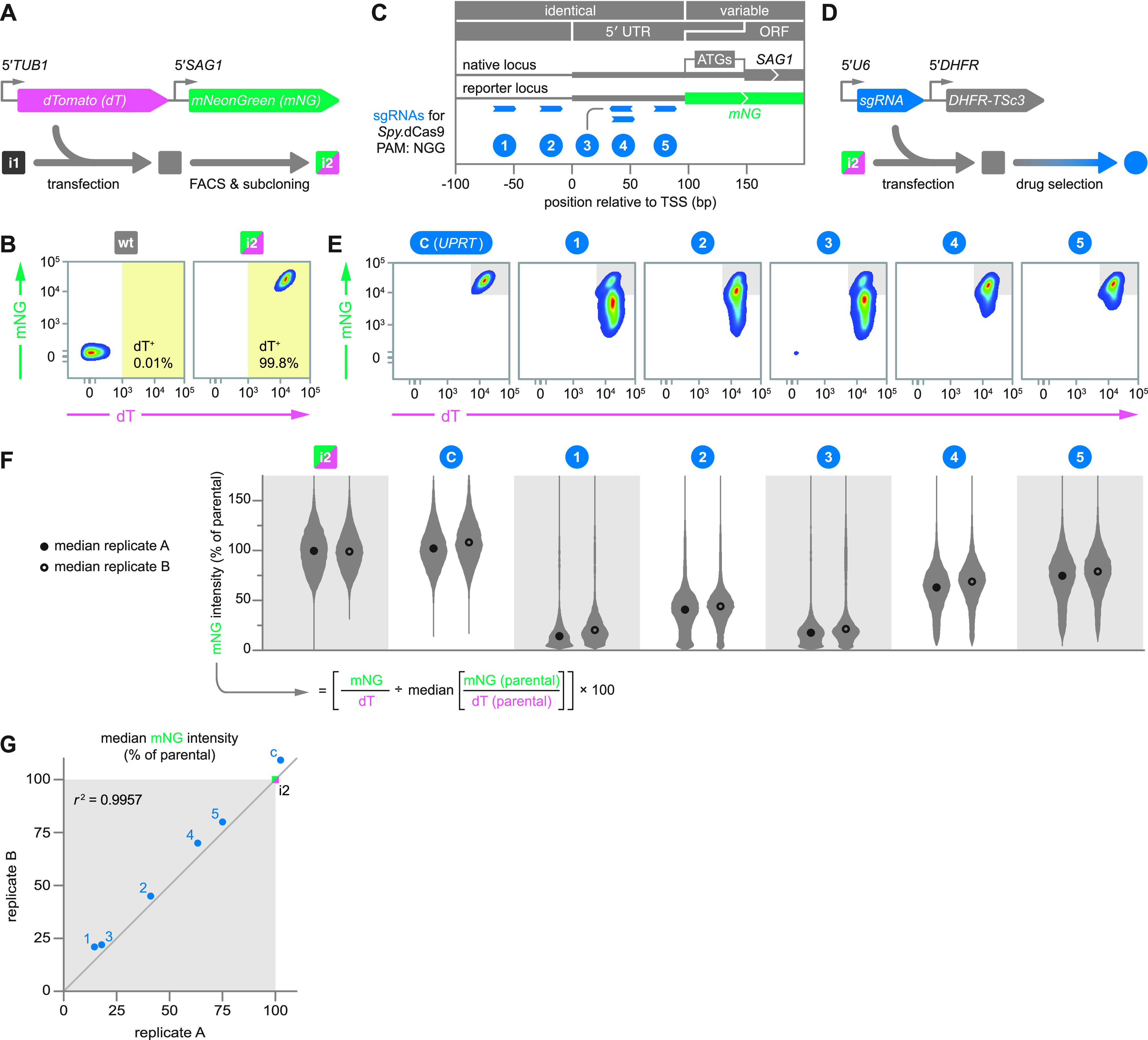
Targeted transcriptional repression using S. pyogenes dCas9. (A) Expression construct and strategy for generating a clonal dual-fluorescence reporter strain (i2) in the *Spy*.dCas9-expressing background (i1). mNG and dT were expressed from copies of constitutively active *Toxoplasma* promoters and 5′ UTRs. FACS, fluorescence-activated cell sorting. (B) Flow cytometry pseudocolor density plots of wt and i2 parasites. Axis values of these and all subsequent flow cytometry plots were biexponentially transformed to provide a more accurate visual representation of fluorescence units in the lower range of the scale; i.e., for low-magnitude values (around 0), the scaling is displayed as if it were linear (see Materials and Methods for details). (C) To-scale diagram showing the target sites (protospacers) and strandedness of five sgRNAs at the *SAG1* and *mNG* loci. sgRNAs were designed for cotargeting these loci within a window from −50 to +95 bp relative to the TSS. The *SAG1* gene model was based on ORF data from the reference annotation (ToxoDB.org, GT1 v.10) and work that previously mapped the dominant TSS (24, 25). We have since appreciated that the experimentally validated 5′ UTR extends past the first ATG to a second in-frame ATG, 51 nt further into the ORF of the reference annotation (37), which explains the truncated 5′ UTR we used for the mNG reporter. PAM, protospacer-adjacent motif. (D) Expression construct and strategy for generating sgRNA-expressing parasite populations in the dual-fluorescence reporter background. A pyrimethamine-resistant allele of the bifunctional dihydrofolate reductase/thymidylate synthase gene (*DHFR-TSc3*) served as a selectable marker. (E) Flow cytometry pseudocolor density plots of drug-selected i2-sgRNA transfectants (replicate A). Parasites were individually transfected with expression cassettes for sgRNAs targeting the *SAG1*/*mNG* loci (sgRNAs 1 to 5) or a control sgRNA (sgRNA C) designed to target the dispensable *UPRT* ORF. Flow cytometry was conducted on day 13 posttransfection, following drug selection for stable construct integration. (F) Violin plots of normalized mNG signal intensities from i2 and drug-selected i2-sgRNA transfectants. Data were collected via flow cytometry on day 13 posttransfection from two independent experiments (replicates A and B). At the bottom is the formula used to normalize the mNG-to-dT intensity ratio for each cell to the median ratio in the parental i2 population. To prevent debris and dead cells from distorting these calculations, we used only dT-positive events, as per the gate shown in panel B. (G) Correlation of median normalized mNG intensities from replicates A and B.

To test the repressive capacity of *Spy*.dCas9 in the context of different sgRNAs, we generated a dual-fluorescence reporter construct to monitor gene expression by flow cytometry. Here, expression of dTomato (dT) and mNeonGreen (mNG) is controlled by two different constitutively active *Toxoplasma* promoters and their associated 5′ untranslated regions (collectively referred to as the upstream region) (Fig. 2A). In the reporter construct, dT expression was driven by the upstream region of the alpha tubulin gene (*TUB1*), while mNG was placed under the control of the upstream region of *SAG1*. Using this construct, we generated a clonal parasite line (i2) in the i1 background that was distinctly fluorescent in both the dT and mNG channels by flow cytometry (Fig. 2A and B). Since the design of this construct, we have appreciated that the experimentally validated 5′ untranslated region (UTR) of *SAG1* extends past the first ATG, 51 nucleotides (nt) further into the open reading frame (ORF) of the reference annotation (37), which explains the truncated 5′ UTR we used for the mNG reporter (Fig. 2C).

The *SAG1* upstream region represented a suitable target for evaluating transcriptional repression, as (i) its high baseline activity provides a broad dynamic range for measuring repression; (ii) transcription initiation activity at the *SAG1* promoter is spatially organized within a sharp peak, with the major transcription start site (TSS) located at −146 bp relative to the ORF (24); and (iii) *SAG1* is largely dispensable to parasites *in vitro* (5), minimizing confounding effects from selective pressure for or against repression. We therefore designed five sgRNAs to uniquely target either DNA strand of the *SAG1* upstream region within −50 to +95 bp of the major TSS (24, 25) (Fig. 2C). We chose this targeting window based on the region previously found to be most effective for CRISPRi in human cells (−50 to +300 bp) (17). Next, we cloned these sgRNAs into independent drug-selectable expression constructs, which were individually transfected into the dual-fluorescence *Spy*.dCas9 reporter strain (Fig. 2D). Following transfection, cells were cultured with pyrimethamine to select for stable construct integration until they lysed host cell monolayers in a regular 2-day cycle. At this point (typically after 13 days), we considered parasites to be drug selected. To assess the effects associated with targeting *Spy*.dCas9 to the *SAG1* upstream region, we measured mNG and dT signal intensities of drug-selected parasites by flow cytometry (Fig. 2E). Parasites receiving any sgRNA targeting the *SAG1* upstream region (sgRNAs 1 to 5) showed diminished mNG signal intensity, while untransfected parasites or those that received a control sgRNA targeting the dispensable *UPRT* locus (sgRNA C) showed no change in fluorescence. The dT signal intensity remained stable across all populations irrespective of sgRNA, corroborating the specificity of the observed effect at the *SAG1* upstream region.

To accurately compare the effects of different sgRNAs, we normalized the mNG-to-dT intensity ratio for each cell to the median ratio in the parental i2 population (Fig. 2F). We then compared the medians of the normalized mNG signals from individual populations and found that the effect of each sgRNA was highly reproducible between independently transfected and drug-selected parasite populations (*r*^2^ = 0.9957), but knockdowns varied between 5.6-fold for sgRNA 1 and 1.3-fold for sgRNA 5, relative to the untransfected i2 strain (Fig. 2F and G). The stereotypical effect of each sgRNA suggests that their variable effects originate from inherent properties such as target site or sequence, rather than variability in transfection or drug selection. Within this small data set, the repressive capacity of an sgRNA largely appeared to be a function of the targeting distance to the gene’s TSS. However, we note that this correlation was imperfect, with sgRNA 2 being less effective than the more distally targeting sgRNAs 1 and 3. The two most effective sgRNAs, sgRNAs 1 and 3, targeted opposite strands, suggesting that repression may be independent of sgRNA orientation on the targeted DNA strand. The proposed importance of TSS proximity and the irrelevance of strandedness for transcriptional repression by *Spy*.dCas9 are consistent with CRISPRi studies in human cell lines (17) and with initial findings in P. yoelii (13). Surveying a more comprehensive array of sgRNAs, however, will be necessary to fully elucidate the rules of repression by *Spy*.dCas9 and to test whether nontemplate strand-targeting sgRNAs are overall more effective, as has been observed in some organisms (11, 26).

### Transcriptional repression by S. pyogenes dCas9 can be stably maintained.

**FIG 3:**
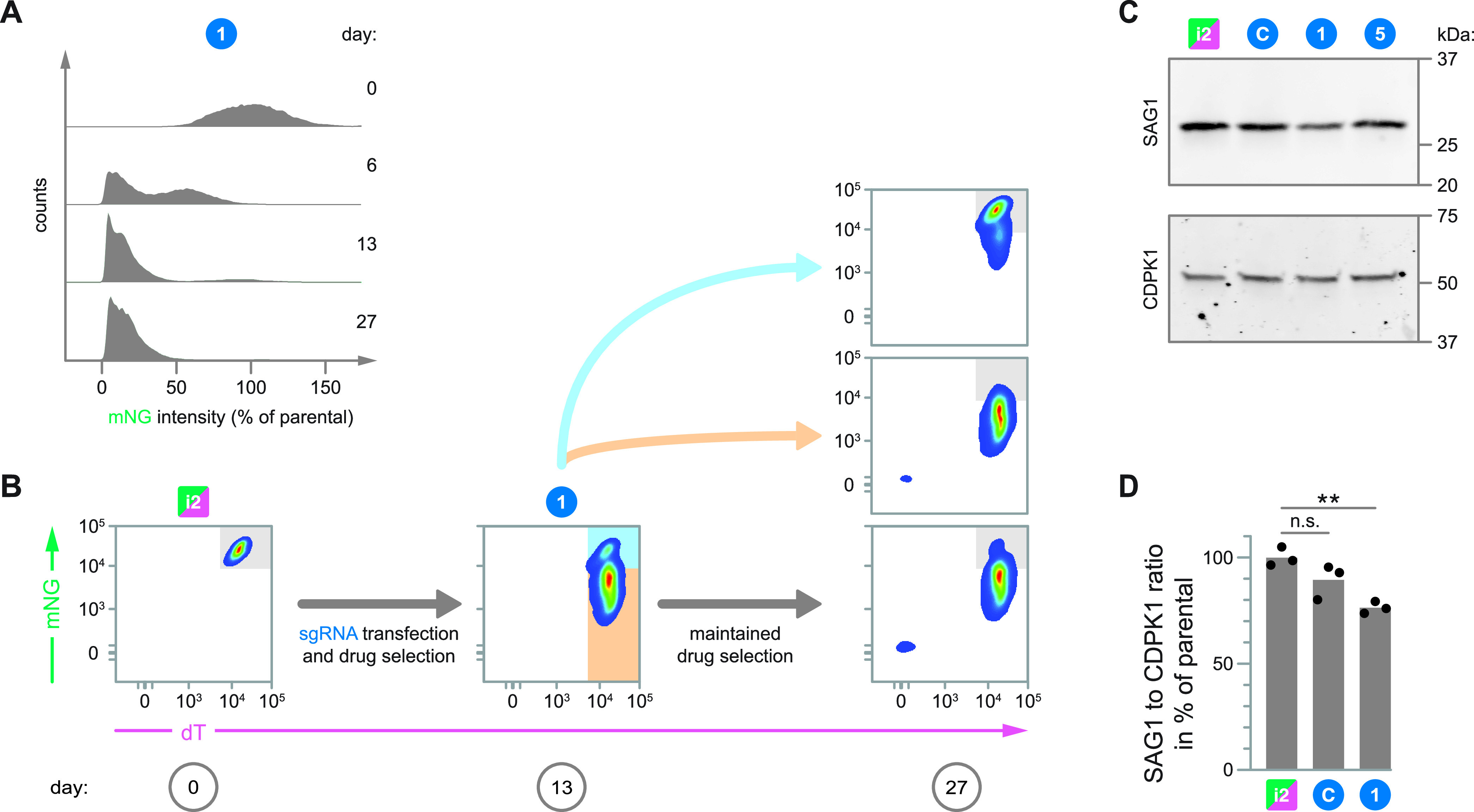
Transcriptional repression by S. pyogenes dCas9 is durable and stably inherited. (A) Density plots of normalized mNG signal intensity for i2 (day 0) and an i2-sgRNA 1-transfectant over the course of 27 days of maintained drug selection. (B) Flow cytometry pseudocolor density plots of i2 pretransfection (left) and on days 13 (middle) and 27 (right) PT with sgRNA 1. The drug-selected population was sorted on day 13 PT using the color-coded sort gates. Sorted and unsorted populations were maintained under drug selection for the sgRNA expression cassette. Samples from panels A and B correspond to replicate A of Fig. 2. (C) Immunoblots probing for SAG1 in lysates from i2 and drug-selected i2-sgRNA transfectants. CDPK1 served as a loading control. (D) Densitometric quantification of immunoblots probing for SAG1 abundance in lysates from i2 and drug-selected i2-sgRNA transfectants. SAG1 abundance was normalized to that of the loading control CDPK1. Data are means for 3 biological replicates. Corresponding blots are shown in Fig. S1. Significance was calculated via an unpaired Student's *t* test. **, *P ≤ *0.01; n.s., nonsignificant (*P > *0.05).

Durable and stable gene repression is crucial to reliably generate measurable phenotypes. To assess the durability of repression by *Spy*.dCas9, we tracked changes in mNG signal intensity of the dual-fluorescence reporter strain by flow cytometry following transfection with an sgRNA expression cassette. Specifically, we assessed samples taken pretransfection as well as 6, 13, and 27 days posttransfection with the most effective sgRNA (sgRNA 1) and following continuous drug selection for its expression cassette (Fig. 3A). Indeed, we observed that the distribution of normalized mNG signal intensities within the population gradually became more homogeneous and highly repressed, with little change occurring past the 13-day mark, demonstrating that *Spy*.dCas9 can induce durable repression.

10.1128/mSphere.00474-21.1 FIG S1 Transcriptional repression by S. pyogenes dCas9 is durable and stably inherited (supplemental to Fig. 3D). Immunoblots probing for SAG1 and CDPK1 in lysates from i2 and drug-selected i2-sgRNA transfectants. CDPK1 served as a loading control. Lysates were prepared from three independently transfected and drug-selected parasite populations. Download FIG S1, EPS file, 1.5 MB. Copyright © 2021 Markus et al. 2021 Markus et al. https://creativecommons.org/licenses/by/4.0/ This content is distributed under the terms of the Creative Commons Attribution 4.0 International license.

Even after drug selection for an sgRNA expression cassette, mNG expression levels can vary over an order of magnitude within a population. To gain insight into this heterogeneity, we used fluorescence-activated cell sorting (FACS) to isolate subpopulations with high or low repression levels from drug-selected parasites that had been transfected with sgRNA 1 (Fig. 3B). Following an additional 14 days of continued culture under drug selection, the sorted populations were again assessed by flow cytometry. At this point, the unsorted population had mostly lost the subpopulation of unrepressed cells that had been present during FACS, while the populations sorted for high or low repression maintained the phenotype for which they were originally sorted. Therefore, the degree of repression is a heritable feature, and the repression heterogeneity within a given population is a function of the transfection and drug selection processes, which may produce a spectrum of sgRNA expression levels across the parasite population.

Having characterized the effects of *Spy*.dCas9 on the expression of the mNG reporter, we sought to determine whether expression of *SAG1* was subject to similar repression by the sgRNAs tested. We examined the lysates from parental and drug-selected parasites by immunoblotting for SAG1 protein (Fig. 3C and D; also, see Fig. S1 in the supplemental material). Indeed, the relative effects of the sgRNAs that were least (sgRNA 5) and most (sgRNA 1) effective at the *mNG* reporter locus were recapitulated at the native *SAG1* locus; however, the overall magnitude of repression at the native *SAG1* locus was more subtle than what was observed for the reporter. Our *mNG* reporter employs a fragment of the *SAG1* upstream region, integrated at an undetermined site within the genome of strain i2. It is likely that the amenability of a given upstream region to repression by *Spy*.dCas9 depends on its broader genomic and epigenomic context, which may explain the differences in repression between the *SAG1* and *mNG* reporter loci. Indeed, such exogenous reporter expression cassettes frequently lack the ability to accurately represent how well gene regulation systems can affect the expression of endogenous genes (13).

### Targeted transcriptional repression using S. thermophilus dCas9.

**FIG 4:**
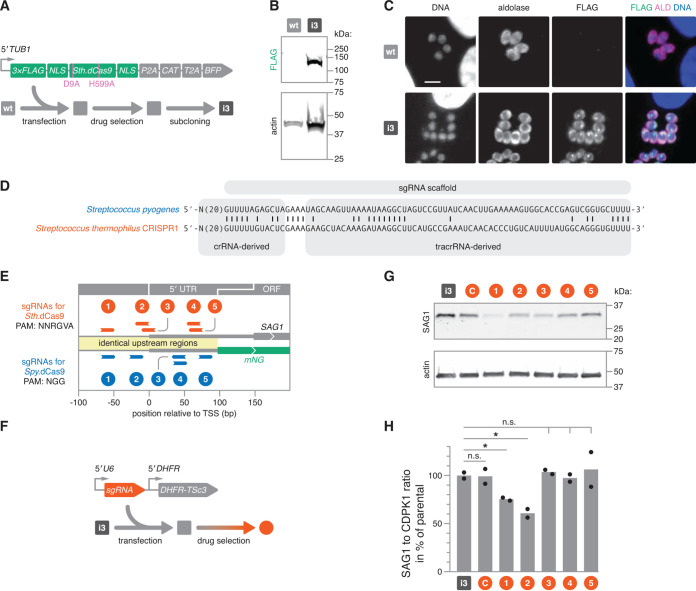
Targeted transcriptional repression using S. thermophilus dCas9. (A) Expression construct and strategy for generating a clonal *Sth*.dCas9-expressing strain. *Sth*.dCas9 was expressed with an N-terminal FLAG tag and transcriptionally linked with resistance and fluorescence markers via viral 2A peptides. Following transfection and chloramphenicol selection of this construct in wt parasites, the resulting population was subcloned by limiting dilution to isolate CRISPRi strain 3 (i3). BFP, blue fluorescent protein; NLS, nuclear localization signal. (B) Immunoblots probing for FLAG and parasite actin. The expected molecular weight of FLAG-tagged *Sth*.dCas9 is 138 kDa. Actin served as a loading control. (C) Representative immunofluorescence images of formaldehyde-fixed intracellular parasites. Cells were stained for FLAG. Parasite aldolase (ALD) provides a cytosolic parasite stain, and Hoechst was used to stain host and parasite DNA. Bar, 5 μm. (D) Engineered sgRNA sequences for Cas9 orthologs from S. pyogenes (33) and from the S. thermophilus CRISPR1 locus (30) which were used in this study. Matching nucleotide positions between the sgRNA scaffolds are indicated. (E) To-scale diagram comparing target sites and strandedness of sgRNAs from the two Cas9 orthologs at the *SAG1* and *mNG* loci. PAMs used for the design of these sgRNAs are indicated. (F) Expression construct and strategy for generating sgRNA-expressing parasite populations in the *Sth*.dCas9 background. (G) Immunoblots probing for SAG1 and parasite actin in lysates from i3 and drug-selected i3-sgRNA transfectants. Actin served as a loading control. (H) Densitometric quantification of immunoblots probing for SAG1 abundance in lysates from i3, and drug-selected i3-sgRNA transfectants. SAG1 abundance was normalized to that of the loading control CDPK1. Data are means for 2 biological replicates. Corresponding blots are shown in Fig. S2. Significance was calculated via an unpaired Student's *t* test. *, *P ≤ *0.05; n.s., nonsignificant (*P > *0.05).

In view of the limited effects of some sgRNAs at the *mNG* reporter locus and the overall minor repression at the native *SAG1* locus, we sought to improve upon the *Spy*.dCas9 system for CRISPRi in *Toxoplasma*. In Mycobacterium tuberculosis, the potency of different dCas9 orthologs can vary substantially (27). In fact, transcriptional repression by *Spy*.dCas9 was outperformed by a smaller dCas9 version derived from the CRISPR1 locus of Streptococcus thermophilus (*Sth*.dCas9), which enabled the first CRISPRi-based screens in M. tuberculosis (27, 28). To test the function of *Sth*.dCas9 in *Toxoplasma*, we generated a *Toxoplasma*-specific *Sth*.dCas9 expression construct that harbors point mutations (D9A and H599A)—analogous to those used to generate *Spy*.dCas9—which abrogate nuclease activity (11, 22) (Fig. 4A). Expression of *Sth*.dCas9 was transcriptionally linked to the chloramphenicol acetyltransferase (CAT) selectable marker and a blue fluorescent protein (BFP) via viral 2A peptides. This design was based on findings from a parallel study that showed that 2A peptides can be used to stably select for *Spy*.Cas9 and *Spy*.dCas9 expression (21). Following transfection of this construct into wt *Toxoplasma* and drug selection for stable integration of the expression cassette, we isolated the clonal CRISPRi strain 3 (i3). This strain expressed full-length *Sth*.dCas9, as observed by immunoblotting (Fig. 4B). Despite carrying identical nuclear localization sequences at corresponding sites, immunofluorescence microscopy for the N-terminal FLAG tag suggested a subcellular localization of *Sth*.dCas9 that was distinct from that observed for *Spy*.dCas9, being predominantly cytosolic with an additional focus within the nucleus (Fig. 4C). We did not pursue further experiments to determine the cause for the alternative localization patterns; however, we hypothesize that these patterns may be due to differences in expression levels and/or preferences in localization that are inert to each protein ortholog. In principle, little dCas9 protein is required to induce transcriptional repression, which warranted the functional assessment of strain i3.

10.1128/mSphere.00474-21.2 FIG S2 Targeted transcriptional repression using S. thermophilus dCas9 (supplemental to Fig. 4H). Immunoblots probing for SAG1 and CDPK1 in lysates from i3 and drug-selected i3-sgRNA transfectants. CDPK1 served as a loading control. Lysates were prepared from two independently transfected and drug-selected parasite populations. Download FIG S2, EPS file, 1.7 MB. Copyright © 2021 Markus et al. 2021 Markus et al. https://creativecommons.org/licenses/by/4.0/ This content is distributed under the terms of the Creative Commons Attribution 4.0 International license.

Cas9 orthologs from S. pyogenes and the S. thermophilus CRISPR1 locus have different protospacer-adjacent-motif (PAM) and sgRNA scaffold requirements, which required the use of different sgRNAs with distinct target sites for each dCas9. While identical in length, the respective sgRNA scaffolds are highly divergent at the nucleotide sequence, sharing only ∼39% sequence identity (Fig. 4D). A previous study demonstrated that these scaffolds are not interchangeable and can enable the fully orthogonal use of CRISPR systems derived from S. pyogenes and the S. thermophilus CRISPR1 locus (28). For this study, we designed all sgRNAs with 20 nucleotides of complementarity (spacer) to a target site (protospacer) immediately adjacent to an appropriate PAM. As consensus PAMs, we used NGG for *Spy*.dCas9 and NNRGVA for *Sth*.dCas9 (27).

We designed five *Sth*.dCas9-specific sgRNAs that targeted the same segment of the *SAG1* upstream region as their *Spy*.dCas9 counterparts (Fig. 4E). We then generated individual sgRNA expression constructs, which we transfected into strain i3 (Fig. 4F). Following drug selection for stable integration of the sgRNA expression cassette into the parasite genome, we measured *SAG1* expression by immunoblotting and observed significant loss of SAG1 protein induced by two of five sgRNAs (Fig. 4G and H; Fig. S2). As with *Spy*.dCas9, sgRNAs closer to the *SAG1* TSS (sgRNAs 1 and 2) tended to be more effective. However, targeting distance from the TSS alone was again insufficient to explain the observed differences in repressive capacity between sgRNAs, as was demonstrated by sgRNA 3. Strikingly, the most effective sgRNA in the *Spy*.dCas9 system and the second most effective sgRNA in the *Sth*.dCas9 system (sgRNAs 1) targeted protospacers offset by only one base pair and with identical strandedness, suggesting that this target site and/or spacer/protospacer sequence is particularly effective for repression of *SAG1* by CRISPRi. This target is located just downstream of the six 27-bp repeats that are required for promoter activity (29) and by inference, appears to be located within the nucleosome-depleted region (24).

Overall, these results suggest that transcriptional repression in *Toxoplasma* can be achieved with both *Sth*.dCas9 and *Spy*.dCas9. Although our study was not designed to directly compare the two constructs, our data suggest that *Sth*.dCas9 may outperform *Spy*.dCas9 in *Toxoplasma*. More comprehensive studies are needed to establish the quantitative difference in performance between the two orthologs, e.g., by targeting multiple genes across a spectrum of expression levels with various sgRNAs, and assessing repression by qRT-PCR. One potential drawback to the implementation of *Sth*.dCas9 is the decreased frequency of compatible PAMs across the genome. Nevertheless, the availability of two orthogonally compatible dCas9 orthologs provides an expanded repertoire of tools for transcriptional regulation and other dCas9-based approaches in *Toxoplasma*.

## DISCUSSION

Catalytically inactive Cas9 orthologs from S. pyogenes and S. thermophilus can act as RNA-guided repressors of transcription in *Toxoplasma*. Targeting these dCas9 orthologs to distinct sites within the *SAG1* upstream region in both its native and reporter context, generated highly reproducible repression levels. Our results suggest that *Sth*.dCas9 may outperform *Spy*.dCas9 in *Toxoplasma*; however, our study was not designed to directly compare them. Consistent with CRISPRi studies previously performed in mammalian cells (17), we found that the distance of the sgRNA target site from the promoter’s TSS is likely critical for the magnitude of repression, although target-to-TSS distance alone cannot fully explain the repressive capacity of a given sgRNA. Repression also appeared to be largely independent of the targeted DNA strand. Therefore, tuning the expression of targeted genes will likely require testing multiple sgRNAs to empirically determine their effects on transcription. In our reporter system, the transcriptionally repressed state was stably inherited and could be maintained at least over the course of several weeks.

The reproducibility and durability of repression, combined with the ability to induce target site-specific degrees of repression are ideal features for generating measurable phenotypes that can be studied in single- or multigene experiments as well as in pooled-screening approaches. Dosing the expression of target genes is an effective tool for characterizing genes in studies of drug mechanism of action or epistasis (reviewed in reference 19). Further work, however, is needed to more comprehensively compare and evaluate CRISPRi using the two dCas9 orthologs and to generate better guidelines for effective sgRNA design.

The two dCas9 orthologs from S. pyogenes and S. thermophilus are fully orthogonal to one another (30); as such, they are capable of targeting distinct sets of nonoverlapping sequences within the same cell. Together, these proteins could constitute the basis of a platform enabling simultaneous transcriptional regulation (11, 31), labeling of distinct genomic loci for microscopy studies (32), or delivery of a range of other activities to genomic regions of interest. With our experimental demonstration of CRISPRi by two orthogonally compatible dCas9 proteins in *Toxoplasma*, we have expanded the inventory of tools for transcriptional manipulation and other dCas9-based approaches in this organism.

Even without the fusion of transcriptionally repressive domains, dCas9 orthologs can modestly reduce the expression of target genes. Defining endogenous transcriptional repressors in *Toxoplasma* may enable the generation of even more effective CRISPRi systems. Additionally, future studies may test whether the potency of less active sgRNAs using the originally conceived sgRNA scaffold (33) can be enhanced by a design with improved sgRNA expression and affinity to dCas9 (32). Given the positional dependency of CRISPRi observed in other systems (17), we recently mapped transcription initiation events across the *Toxoplasma* genome and defined high-confidence TSSs for the majority of protein-coding genes (24), which will be key in selecting effective sgRNAs for use in future CRISPRi studies. Lastly, developing tightly controlled, inducible dCas9 systems for achieving immediate and maximal gene knockdown following drug selection for sgRNA expression cassettes will likely be beneficial for a range of CRISPRi applications in *Toxoplasma*. Such systems could also allow reversible gene knockdowns (11) and studies of gene function within temporally defined processes.

## MATERIALS AND METHODS

### Parasites and host cells.

*Toxoplasma* tachyzoites from the wild-type strain RH and derived strains were maintained at 37°C with 5% CO_2_, growing in human foreskin fibroblasts (HFFs; ATCC SCRC-1041) cultured in Dulbecco’s modified Eagle’s medium (DMEM; Gibco) supplemented with 3% or 10% heat-inactivated fetal bovine serum and 10 μg/ml gentamicin (Thermo Fisher Scientific). Transgenic parasites were obtained by electroporation of constructs into cells and selection with 40 μM chloramphenicol (Sigma-Aldrich) or 3 μM pyrimethamine (Sigma-Aldrich). Clones were isolated by limiting dilution.

### Transfection.

Freshly egressed parasites were passed through a polycarbonate filter with a 3-μm pore size (Whatman), then washed, and resuspended in Cytomix (34) (10 mM KPO_4_, 120 mM KCl, 150 mM CaCl_2_, 5 mM MgCl_2_, 25 mM HEPES, 2 mM EDTA) to 8 × 10^7^ cells/ml. Two hundred forty-five microliters of the parasite suspension was combined with 50 μg of previously linearized plasmid in 155 μl Cytomix, supplemented with 2 mM ATP and 5 mM glutathione (GSH), to a final volume of 400 μl. Parasites were electroporated in 4-mm-gap cuvettes (BTX Harvard Apparatus model no. 640) in an Electro Square Porator (BTX Harvard Apparatus) set to 1.7 kV, for two 176-μs pulses at 100-ms intervals.

### Immunoblot and densitometry.

Blots shown in Fig. 1 and 4 were generated with the following protocol. Freshly egressed parasites were filtered and washed in phosphate-buffered saline (PBS) before lysis in 1× Laemmli buffer (4% SDS, 20% glycerol, 5% 2-mercaptoethanol, 0.02% bromophenol blue, 120 mM Tris-HCl [pH 6.8]). 2-Mercaptoethanol was not included when probing for SAG1. Samples were heated to 100°C for 5 min prior to resolving by SDS-PAGE. After transferring the separated proteins onto nitrocellulose at a constant 25 V overnight, membranes were blocked for 1 h at room temperature in PBS with 5% (wt/vol) nonfat dry milk. Immunoblots were probed, as indicated, with mouse anti-FLAG (M2; Sigma-Aldrich) diluted 1:5,000, rabbit anti-actin (TgACT1) (35) diluted 1:10,000, or mouse monoclonal anti-SAG1 (clone DG52) (25) diluted 1:1,000. The signal was detected using 1:20,000 dilutions of IRDye 800CW-conjugated goat anti-mouse IgG and IRDye 680RD-conjugated donkey anti-rabbit IgG (LI-COR Biosciences) on an Odyssey infrared imager (LI-COR Biosciences). The blots shown in Fig. 3 and in Fig. S1 and S2 were generated with the following protocol. Freshly egressed parasites were filtered and washed in PBS before lysis in 1× lysis buffer (1% Igepal 630 CA, 150 mM NaCl, 20 mM Tris-HCl [pH 7.5], 1× Halt protease inhibitors [Thermo Scientific]). Samples were incubated on ice for 10 min, and lysate was clarified by centrifugation for 5 min at 20,000 × *g*. Protein concentrations were measured using a DC protein assay (Bio-Rad) prior to diluting and denaturing in 1× Laemmli buffer without reducing reagents for 10 min at 37°C. Samples were resolved on a 4-to-15% SDS-PAGE gel and transferred onto nitrocellulose at a constant 90 V for 1 h. Membranes were blocked for 1 h at room temperature in PBS with 5% (wt/vol) nonfat dry milk. Immunoblots were probed as indicated with mouse monoclonal anti-SAG1 (clone DG52) diluted 1:1,1000 or guinea pig anti-CDPK1 (Covance) (36) diluted 1:50,000. The signal was detected using 1:10,000 dilutions of IRDye 800CW conjugated donkey anti-mouse IgG and IRDye 680RD conjugated donkey anti-guinea pig IgG (LI-COR Biosciences) on an Odyssey infrared imager (LI-COR Biosciences). Densitometric quantification was performed using Image Studio Light (v. 5.2.5; LI-COR Biosciences).

### Immunofluorescence staining and microscopy.

Intracellular parasites were fixed on glass coverslips at 4°C with 4% formaldehyde for 10 min. Formaldehyde-fixed samples were permeabilized with 0.25% Triton X-100 in PBS for 8 min. Mouse monoclonal antibodies were used to detect SAG1 (clone DG52) (25), and FLAG-tagged dCas9 orthologs (clone M2; Sigma-Aldrich). Rabbit polyclonal serum was used to detect aldolase (WU1614) (23). Primary antibodies were detected with Alexa-Fluor-labeled secondary antibodies. Nuclei were stained with Hoechst (Santa Cruz) and coverslips were mounted in Prolong Diamond (Thermo Fisher). Images were acquired using an Eclipse Ti epifluorescence microscope (Nikon) using the NIS elements imaging software. Adobe Photoshop was used for image processing.

### Flow cytometry.

Freshly egressed parasites were passed through a polycarbonate filter with a 3-μm pore size (Whatman), pelleted at 1,000 × *g*, and resuspended in PBS before addition of an equal volume of 8% formaldehyde in PBS. Following incubation for 15 min at room temperature, parasites were pelleted and washed twice before being resuspended in PBS. Fluorescence from mNeonGreen and dTomato was detected on a FACSCanto II (BD Biosciences). Flow cytometry data were acquired with FACSDiva software (BD Biosciences) and analyzed using FlowJo. For the display of all pseudocolor density plots, a biexponential scaling was used with the following parameters: extra negative decades = 0, width basis = +4.40, positive decades = −31.6. To exclude debris and dead cells from the calculation of normalized mNG signal intensities, we gated on dT-positive events, as per the gate shown in Fig. 2B.

### sgRNA design.

Table 1 details all sgRNAs and protospacers that were used in this study. Custom scripts were used to assess potential off-target sites of S. pyogenes and S. thermophilus dCas9 spacers. Only protospacers that diverged by at least three nucleotides from any secondary genomic target within the GT1 genome (ToxoDB version 10) were considered uniquely targeting and used in this study. To aid RNA expression from the *U6* promoter, a guanine nucleotide was added to the 5′ end of sgRNAs that did not already have one at the 5′-most position. Spacer DNA oligonucleotides were synthesized by IDT and cloned by Gibson Assembly into the respective BsaI-digested sgRNA expression constructs.

**TABLE 1:** sgRNAs used in this study

sgRNA scaffold	Designation	Target^*a*^	Protospacer–PAM (5′-3′)
S. pyogenes	sgRNA C	*UPRT* CDS	GGTCGTTTGTCGAATATCGG–AGG
	sgRNA 1	*SAG1* upstream region	GCCGCACAATGTGCACCTGT–AGG
	sgRNA 2	*SAG1* upstream region	GATTCTCACTGTTCTCGGCA–AGG
	sgRNA 3	*SAG1* upstream region	AAGAACGGAAAGCTGCACAA–CGG
	sgRNA 4	*SAG1* upstream region	GTGCAGCTTTCCGTTCTTCT–CGG
	sgRNA 5	*SAG1* upstream region	GTCATTGTCGTGTAAACACA–CGG
S. thermophilus (CRISPR1)	sgRNA C	*UPRT* CDS	AAAGTACACGACTGAGAGTT–CGAGAA
	sgRNA 1	*SAG1* upstream region	TGCCGCACAATGTGCACCTG–TAGGAA
	sgRNA 2	*SAG1* upstream region	TCGGCCCTTGCCGAGAACAG–TGAGAA
	sgRNA 3	*SAG1* upstream region	CTCCGGTCGTCGGCCCTTGC–CGAGAA
	sgRNA4	*SAG1* upstream region	GACACATGTGACACAACCGA–GAAGAA
	sgRNA5	*SAG1* upstream region	AATGACACATGTGACACAAC–CGAGAA

a CDS, coding sequence.

### Plasmids.

Table 2 details all plasmids and their use in this study. The map of the construct used for generating dually fluorescent cells can be found in the GenBank database via the indicated accession number. For the dual-fluorescence reporter construct, we derived the *SAG1* and *TUB1* upstream regions from sequences in the *Toxoplasma* GT1 genome reference (ToxoDB v.54). A 757-bp fragment from chromosome (Chr) VIII, between 2659698 and 2660454, was used as the SAG1 5′ UTR. A 498-bp fragment from Chr XI, between 5217584 and 5218081, was used as the *TUB1* 5′ UTR.

**TABLE 2:** Plasmids used in this study

Plasmid name	Description	Addgene no.	GenBank no.
pTUB1-Spy.dCas9-CAT-U6-sgRNA(decoy)	Used to generate *Spy*.dCas9 cells (strain i1)	171089	
pSAG1-mNeonGreen_TUB1-dTomato	Used to generate dual-fluorescence reporter cells (strain i2)		MZ090944
pTUB1-Sth.dCas9-P2A-CAT-T2A-TagBFP	Used to generate *Sth*.dCas9 cells (strain i3)	171090	
pU6-Universal	Recipient plasmid for cloning S. pyogenes spacers via BsaI restriction sites (2)	52694	
pU6-Universal.Sth.dCas9	Recipient plasmid for cloning S. thermophilus spacers via BsaI restriction sites	171088	

### Data availability.

The plasmids for generating Toxoplasma stably expressing *Spy.*dCas9 and *Sth.*dCas9, as well as the universal recipient plasmids for Cas9 spacers with specific S. pyogenes and S. thermophilus sgRNA scaffolds, are available from Addgene. The dual-fluorescence reporter construct is available upon request and its sequence is provided under GenBank no. MZ090944.
